# Phase separation in innate immune response and inflammation-related diseases

**DOI:** 10.3389/fimmu.2023.1086192

**Published:** 2023-02-13

**Authors:** Huihui Ma, Mingxi Liu, Rao Fu, Jia Feng, Haoran Ren, Jingyan Cao, Ming Shi

**Affiliations:** ^1^ School of Life Science and Technology, Harbin Institute of Technology, Harbin, China; ^2^ Department of Medical Oncology, Harbin Medical University Cancer Hospital, Harbin, China

**Keywords:** innate immune response, phase separation, intrinsic disorder, inflammatory response, spatiotemporal control

## Abstract

Inflammation induced by nonspecific pathogenic or endogenous danger signals is an essential mechanism of innate immune response. The innate immune responses are rapidly triggered by conserved germline-encoded receptors that recognize broad patterns indicative of danger, with subsequent signal amplification by modular effectors, which have been the subject of intense investigation for many years. Until recently, however, the critical role of intrinsic disorder-driven phase separation in facilitating innate immune responses went largely unappreciated. In this review, we discuss emerging evidences that many innate immune receptors, effectors, and/or interactors function as “all-or-nothing” switch-like hubs to stimulate acute and chronic inflammation. By concentrating or relegating modular signaling components to phase-separated compartments, cells construct flexible and spatiotemporal distributions of key signaling events to ensure rapid and effective immune responses to a myriad of potentially harmful stimuli.

## Introduction

Innate immune system is the first line of host defense against pathogens such as viruses and bacteria. The innate immune signaling is triggered when exogenous pathogens (Pathogen-Associated Molecular Patterns, PAMPs) and endogenous injury-related molecules (Damage-Associated Molecular Patterns, DAMPs) are recognized by cell-surface or cytosolic pattern recognition receptors (PRRs). Activation of immunosensors triggers inflammatory response through the production of interferon and proinflammatory cytokines (1–4).Upon stimulation, the PRRs recruit adaptors and effectors to form higher-order assemblies that act as hub platform to perform signal transduction and signal amplification functions (1, 5, 6).

To trigger the innate immune response, the innate immune cells use germline-encoded PRRs that are located on the cell surface or in various intracellular compartments to detect pathogen or danger-associated chemical patterns (7, 8). Multiple PRRs types are present on these immune cells, such as Toll-like receptors (TLR), retinoic acid-induced gene I-like receptors (RLR), nucleotide oligomeric domain-like receptors (NLR, also known as NACHT, LRR, and PYD domain proteins), and cytoplasmic DNA sensors (8), also with melanoma 2 (AIM2)-like receptors (ALRs) and C-type lectin (CLRs) (9). Phase separation activities have been increasingly investigated in the regulation of innate immune response. The principal constituents and mediators of Liquid- Liquid phase separation (LLPS) are proteins and nucleic acids (RNA and DNA) (10). For example, viral nucleocapsid proteins of respiratory syncytial virus (RSV) (11), measles virus (MeV) (12) and highly infectious Severe Acute Respiratory Syndrome Coronavirus 2 (SARS-CoV-2) (13) can induce phase separation to participate in host signaling transduction. Proteins containing modular domains or inherently disordered regions (IDRs) may trigger the formation of membraneless condensates by dynamic protein-protein interactions, which is a common strategy for signalosomes to carry out key signaling and effector functions in innate immunity and inflammation (14, 15).

A substantial number of the hub proteins that function in the network of innate immune responses are intrinsically disordered proteins (IDPs) or intrinsically disordered region-(IDR) containing proteins, which can induce phase separation (16, 17). The formation of LLPS is based on the capability of intrinsically disordered protein/intrinsically disordered protein regions (IDPs/IDPRs) for polyvalent stochastic interactions (17, 18). Recent structural studies and parallel bioinformatics reveal that phase separation of proteins are frequently involved in spatiotemporal control of cellular innate immune and inflammatory signaling (19, 20). The nucleocapsid protein of SARS-CoV-2 is shown to be the primary structural protein of virions. The nucleocapsid protein engages in robust LLPS after binding to viral RNA to enhance NF-kB activation (21). In addition to innate immune responses, LLPS also mediate signal transduction of adaptive immune responses. T cell receptor (TCR) signaling is essential for T cell activation. On the surface of T cells, T cell clusters are formed by transmembrane receptors (TCR, CD28 and PD-1), tyrosine kinases (LCK and ZAP70), adaptor proteins (LAT and GRB2), and various enzymes (SOS1 and PLCγ1). The multivalent interaction among LAT, GRB2 and SOS1 is mediated by LLPS to form T cell micro-clusters (22). Phase separation has emerged as a key factor in many processes, including chromatin assembly (23) and B-cell lymphogenesis (24). In this review, we will highlight the flexible spatiotemporal control mechanisms and the significance of IDPs/IDRs-mediated phase separation in innate immune and inflammatory pathways.

## cGAS-DNA phase separation enhances innate immune responses to cytosolic DNA

**Figure 1:**
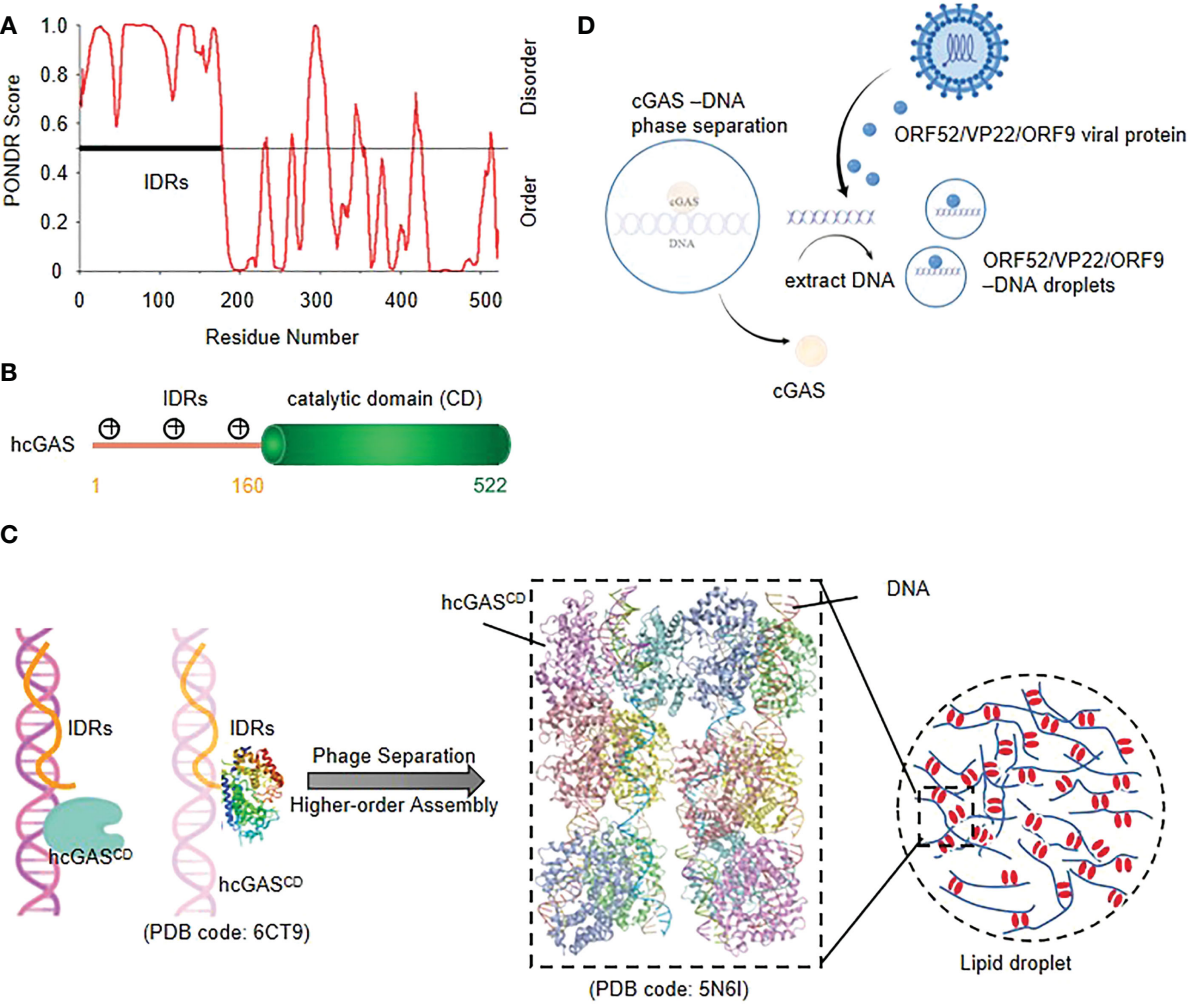
cGAS-DNA phase separation enhances innate immune responses to cytosolic DNA **(A)** Structural prediction of cGAS using PONDR **(B)** Schematic of the domain structure of human cGAS.**(C)** Model of cGAS-DNA phase separation. The IDRs of Nterminal cGAS nonspecifically bind with DNA, and then a robust cGAS-DNA phase separation and higher-order assembly are induced. **(D)** viral proteins restrict cGAS-DNA condensates *in vitro* and in cells; ORF52/VP22 virus can disrupt the cGAS-DNA condensates through extracting the DNA to form new droplets with DNA in order to evade immune surveillance.

Cyclic GMP–AMP synthase (cGAS) is a sensor protein that initiates inflammation in response to cytosolic DNA, a molecular pattern indicative of intracellular pathogens or endogenous damage (25–28). The full length of cGAS consists of a positively charged disordered N-terminal and a structured C-terminal residue (core cGAS) with a nucleotide transferase domain (**Figure 1A**). The high density positively charged residues in the N-terminal domain and three identified DNA binding sites in the C-terminal domain provide the structural basis for the polyvalent interaction between cGAS and DNA (29) (**Figure 1B**).

Upon binding to dsDNA, cGAS dimerizes, assembles into liquid droplets, and is activated to catalyze the synthesis of cyclic GMP-AMP (cGAMP), which then activates the downstream Type I interferon and NF-κB signaling pathway by binding to adaptor protein STING as a second messenger (30). Studies have shown that cGAS and DNA form liquid condensates in a concentration-dependent manner (29). Activated cGAS molecules form supramolecular cytosolic foci that co-localize with DNA, which is a clue that phase separation might underly function (25) (**Figure 1C**).

The cGAS-STING pathway plays an essential role in host defensing against various DNA viruses, while virus explores series of immune evasion strategies against the sensing (31). It has been reported that virus-derived ORF 52, VP22 (32), KicGAS (30) and ORF9 (33) can extract DNA molecules from cGAS-DNA droplets and bind to itself to form new droplets, which leads to the dispersion of cGAS-DNA droplets or reduces the DNA-mediated liquid phase separation of cGAS-DNA, thereby alleviating the cGAS-STING pathway and resulting in immune dysregulation (31) (**Figure 1D**).

The cGAS-DNA phase separation enhances the immune response by inhibiting the activity of TREX1 nuclease, a cytoplasmic ER-related DNA 3’→5’ exonuclease that prevents chronic cGAS activation by degrading cytoplasmic DNA (34). Recent study has confirmed that cGAS-DNA droplets isolate TREX1 to prevent DNA from being degraded and also set barriers to autointegration factor 1 (BAF) from the droplet to prevent improper innate immune signal transduction (35).

## Phase separation of DDX3X decides the cell fate under stress

**Figure 2:**
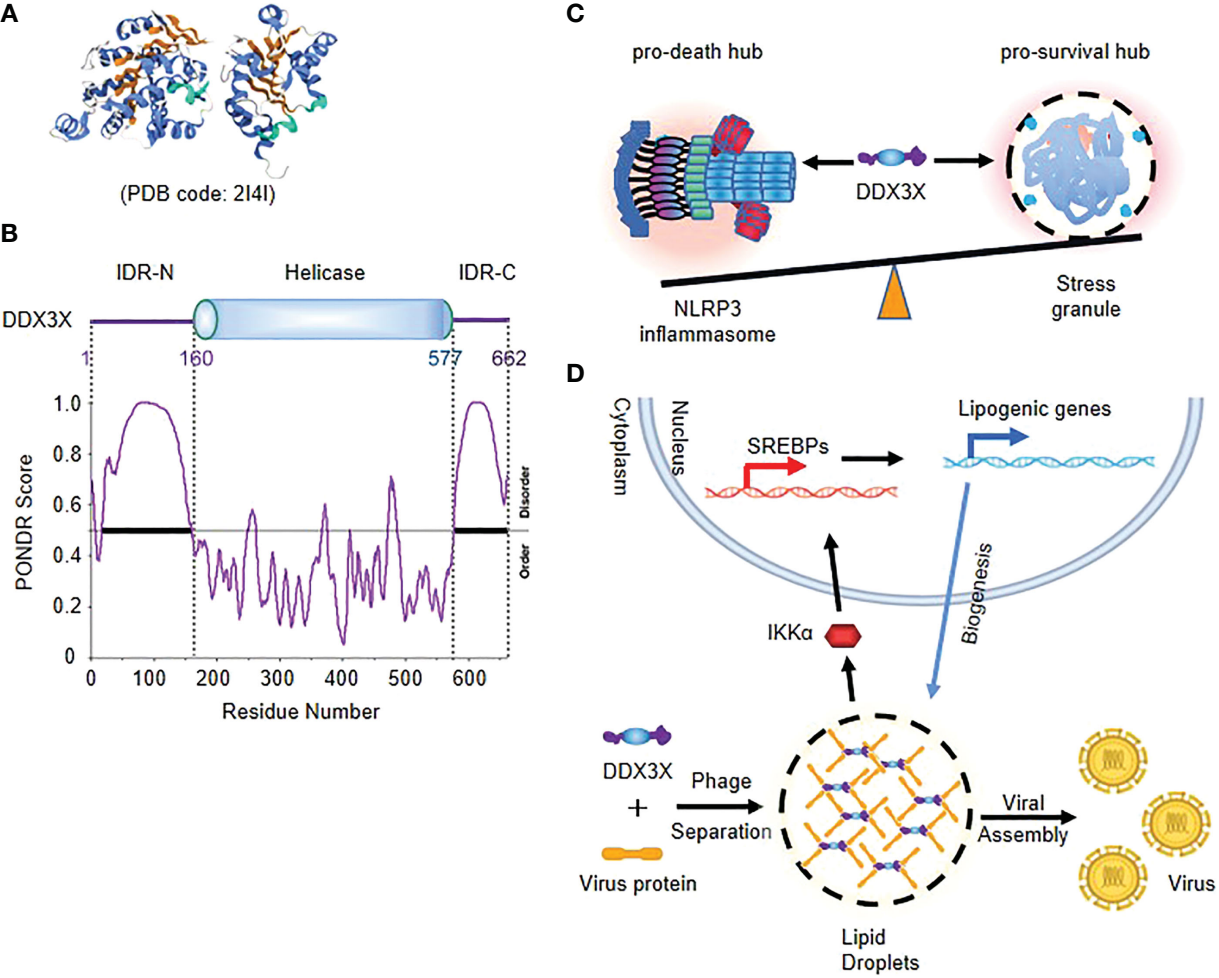
Phase separation of DDX3X decides the cell fate under stress **(A)** Crystal structure of helicase core (V168-G582) of DDX3X. **(B)** The schematic of the domain structure and the intrinsic disorder profile of human DDX3X obtained by PONDR^®^ are shown. **(C)** Model of competition of DDX3X between the pro-survival stress granules and the pyroptotic NLRP3 inflammasome activation. **(D)** Model of DDX3X-enhanced lipogenesis and viral assembly. IDR of DDX3X facilitates the translocation of virus proteins to the liquid droplet surface of DDX3X granules, and leads to IKK-α activation and SREBP-mediated elevated expression of lipogenic genes.

The DEAD-box RNA helicase DDX3X is involved in multiple aspects of RNA metabolism, including RNA splicing, transcription initiation and the assembly of stress particles (36, 37). The structure of helicase core (V168-G582) of DDX3X has been studied by protein crystallography (38) (**Figure 2A**). The N- and C-terminal regions of DDX3X are predicted to be highly disordered by PONDR^®^VSL2 (**Figure 2B**). Biochemical analysis shows that DDX3X is a pivotal component of stress granules (SGs) independent of its RNA helicase activity (39, 40). IDRs in N-terminal of DDX3X can undergo LLPS *in vitro*, and acetylome analysis shows that IDR in N-terminal of DDX3X is a substrate of deacetylase HDAC6, and deacetylation of the IDR by HDAC6 promotes LLPS and assembly of SGs in response to stress, while its acetylation at multiple lysine residues largely impairs the liquid droplet formation (41).

Stress granules are cytosolic compartments formed by cells in the face of various stress conditions, which enable cells to survive under various pressures (42). A recent study suggests that stress granule protein DDX3X serves as a signaling hub that forms in response to various stressors, and the DDX3X molecules are competed by stress granules and the NLRP3 inflammasome to coordinate the activation of innate immune responses and subsequent cell-fate decisions (42, 43). The cytoplasmic stress granule protein DDX3X is identified as an NLRP3 interactor and drives activation of NLRP3 inflammasome independent of RNA helicase activity. The Induction of stress granules sequesters DDX3X, and thereby specifically inhibits NLRP3 inflammasome activation, ASC speck formation and pyroptosis. DDX3X acts as a co-competitive factor for the formation of stress particles and the activation of NLRP3 inflammasomes, and in the binding process, DDX3X enables cells to understand stress signals and determine their fate (**Figure 2C**) (34, 35).

DDX3X participates in the regulation of various viral life cycles and also act as a master regulator in virus-induced cell fate decision. Virus utilizes DDX3X granules as platforms for lipid metabolism and viral assembly (44, 45). DDX3X responds to cellular stress primarily by participating in SG formation that is also a type of LLPS. In productive virus infection, DDX3X facilitates the translocation of virus proteins to the liquid droplet surface of DDX3X granules through initiating its dynamic associations with virus core proteins, which is crucial for lipogenesis and viral assembly (45–47). Moreover, Sannula Kesavardhana et al. showed that DDX3X interferes with the activation of NLRP3 inflammasome by forming SG during IAV infection, thus preventing immune escape from IAV (48) (**Figure 2D**).

In sexual dimorphic helicases DDX3X and DDX3Y, DDX3Y was found to be more droplet oriented than DDX3X, because of the difference in IDR1 sequence. This difference also results in DDX3Y having a stronger ability to aggregate FUS than DDX3X (49). The FET family of proteins, consisting of FUS (TLS), EWS (EWSR1), and TAF15, participates in phase transitions at RNA storage sites and assembles into higher-order structures through RNA stimulation, functions that are impaired in humans and contribute to disease. RNA repeat amplification can also lead to abnormal function, and the repeated RNA sites can capture RNA-binding proteins and cause their loss of function (50). Increased cytoplasmic FUS concentration leads to increased recruitment of stress granules, which leads to neurodegenerative disease.

## Phase separation links with dynamic spatiotemporal regulation of innate immune response

**Figure 3:**
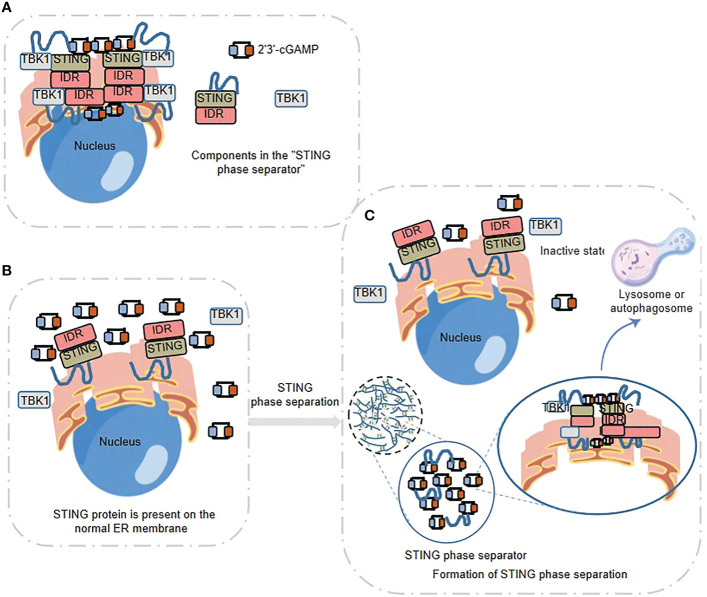
STING phase separator-mediated spatiotemporal regulation of innate immune response **(A)** STING Phase Separator. 2’3’ -cGAMP- STING -TBK1 can be aggregated to form the STING phase separator. **(B)** STING, 2’3’ -cGAMP and TBK1 are present on the normal ER membrane. **(C)** Formation of STING phase separation. The “STING phase separator” comparts the inactive STING-TBK1 (also including 2 ‘3’ -cGAMP) and prevents the over-activation of innate immunity, which finally can be broken down by lysosomes or autophagosomes.

STING is a 4 times transmembrane protein distributed in the endoplasmic reticulum (ER). After binding with 2’3’ - cGAMP, it needs to undergo transport, leaving the ER, passing through the Golgi apparatus, and finally arriving at the small membrane vesicles derived from the Golgi apparatus. The endoplasmic reticulum cubic membrane structure generated by STING phase separation can “sequester” STING-TBK1 and the key transcription factor IRF3 in space, and then negatively regulate the cGAS-STING pathway. Therefore, this cubic membrane structure of ER triggered by high intracellular concentration of 2’3’ - cGAMP, promoted by Mn2+, and formed by phase separation of STING protein is named “STING phase-separator”(**Figure 3A**), which is used to separate inactive STING-TBK1(**Figures 3B, C**). Thus, the STING phase-separator prevents overactivation of innate immunity at a new level (30, 51) (**Figure 3**).

Neurofibroma protein 2 (NF2), a classical tumor suppressor that controls tumor growth and regulates angiogenesis in the tumor microenvironment, has been reported to regulate cGAS-STING signaling through NF2 mutation mediated-LLPS (52–54). NF2 localizes to plasma membranes, cell tight junctions and the cytoskeleton to mediate cell-to-cell signaling, including the Hippo pathway (55). NF2 positively regulates the innate immune response by affecting the activity of the major effector of Hippo pathway-YAP (56, 57). While a patient-derived point mutant of NF2 FERM domain (NF2m) was found to strongly inhibit nucleic acid recognition and subsequent anti-tumor immunity (54). The NF2m-mediated LLPS formation that initiated by the nucleic acid recognition and activation of IRF3, in turn attenuates nucleic acid sensing signals and cGAS-STING signals through acting as an independent docking platform to deactivate TBK1 (54).

Innate sensor NLRP6 has been reported to regulate anti-viral immune response through phase separation-induced inflammasome activation (58). The polybasic regions of NLRP6 are required for phase separation and inflammasome activation. NLRP6 undergoes LLPS to form a membraneless compartment in the cytosol upon binding dsRNA, which serves as a starting point and signaling hub for multiple pathways. NLRP6 condensates activate IFN signaling through recruitment of ASC and activation of caspase-1, and the NLRP6 ligand further promotes the NLRP6 LLPS through leading DHX15/dsRNA into the NLRP6 condensates. Additionally, the K350–354A mutation of NLRP6 impairs multivalent interactions of NLRP6, and NLRP6-mediated inflammasome activation, and thus reduces cell death in immortalized bone marrow-derived macrophages, which suggests that LLPS is an important driving force for higher-order assembly of NLRP6 inflammasome (30, 58, 59).

## Phase separation of autophagy factors balances inflammation

Autophagy is a general homeostatic process, which has been extensively linked to the regulation of innate immune signaling pathways and makes a pivotal contribution to cell autonomous control of inflammation (60). The autophagy pathway has two main specialized physiological functions, the quality control process of selective mitochondrial autophagy (Mitophagy) and the defensive process of exogenous pathogen-engulfment (Xenophagy). Both processes have been demonstrated to control the activation of inflammasomes to limit inflammation (60, 61).

To prevent the excessive inflammasome activation, autophagy deploys an indirect mode of suppression by breaking down the damaged or irreversibly depolarized mitochondria, or has a direct suppressive effect by taking individual inflammasome components as substrates for autophagic degradation (62, 63). Additionally, autophagy components have also been reported to facilitate unconventional secretion of mature IL-1β by engulfment of the autophagic membrane (64). Thus, autophagy seems to play a balancing act of supporting productive inflammatory factors and preventing excessive inflammatory responses.

Liquid-liquid phase separation plays important roles in different steps of autophagy (65). In the process of phase separation mediating autophagy substrates assembly, autophagy cargo receptor p62/SQSTM1 sequesters intracellular misfolded, ubiquitin-positive proteins and mediates cargo delivery for their selective autophagic degradation (66–68). Depending on its self-oligomerization and ubiquitin-binding ability, p62 forms membraneless phase-separated condensates with polyubiquitin, which is critical for its function as a cargo receptor (68, 69).

**Figure 4:**
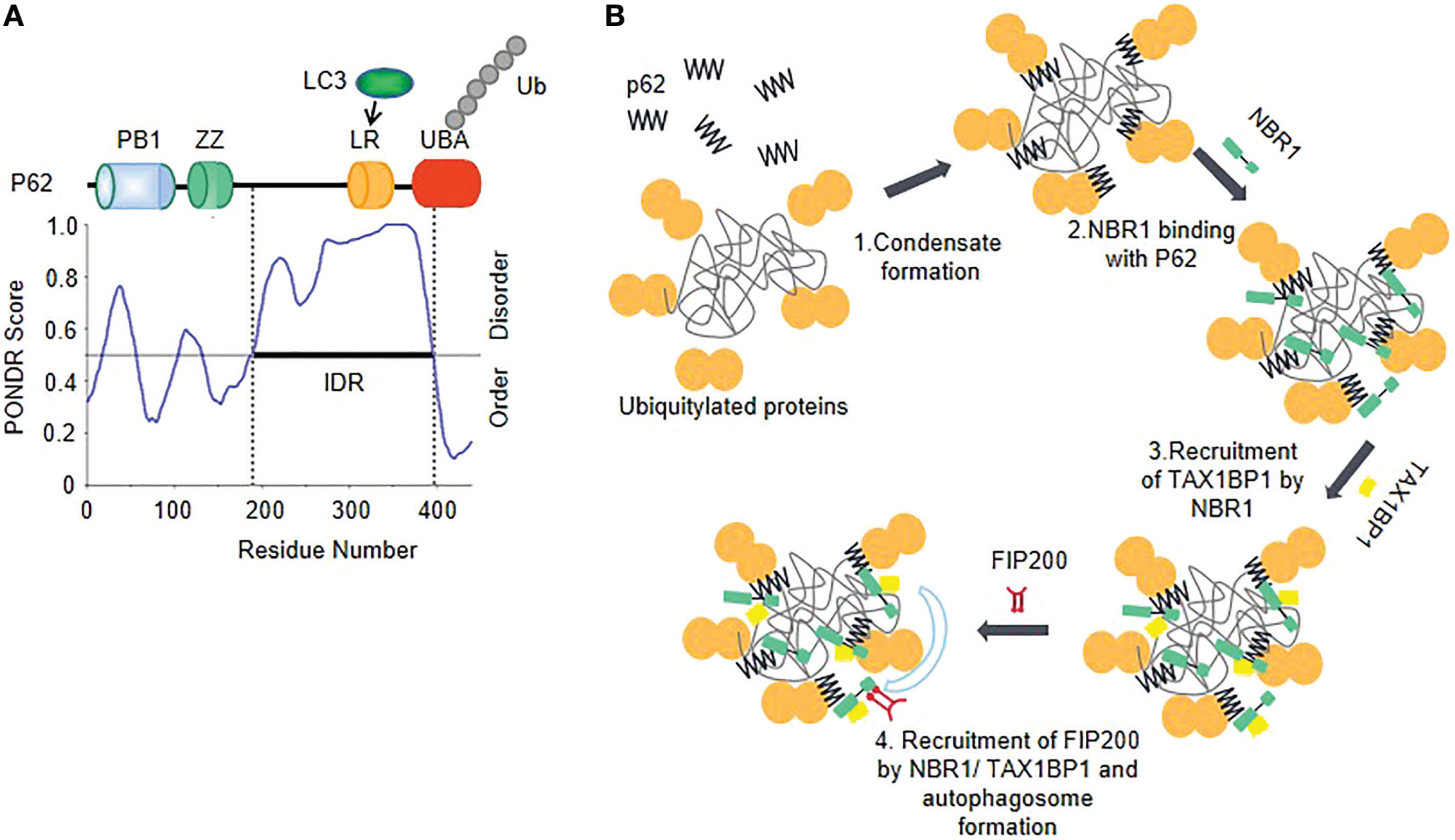
Phase separation of autophagy factors balances inflammation **(A)** The schematic of the domain structure and the intrinsic disorder profile of P62 obtained from PONDR are shown. **(B)** Model of cargo receptors’ interplay in autophagy initiation. Oligomerized p62 and polyubiquitin chains interact with each other to trigger their phase separation and start forming p62 bodies. TAX1BP1 is recruited by NBR-1 which promotes p62 phase separation and initiates FIP200 recruitment.

The IDR has been identified in P62, and the polyubiquitination of P62 further enhances LLPS (66, 68)(**Figure 4A**). P62 recruits the ULK1 subunit FIP200 to the membraneless ubiquitin-positive condensates through the interaction between the IDRs (residues 326-380) of p62 and FIP200-CT (70), which could serve as a platform to initiate autophagosome biogenesis. The IDRs-mediated interaction may promote autophagosome nucleation *via* enhancing the activation of ULK1. Simultaneously, the ULK1 activation triggers negative-feedback control of STING activity to prevent the sustained innate immune signaling (71).

A selective autophagy cargo receptor NBR1 plays an important role in promoting the formation of ubiquitin condensates by directly interacting with the N-terminal PB1 domain of P62. NBR1 introduces its high-affinity ubiquitin-associated (UBA) domain into P62 filaments, thereby facilitating efficient cargo aggregation of P62 (69, 72). NBR1 recruits a third receptor TAX1BP1, which can drive robust FIP200 recruitment, so as to initiate autophagosome formation (72) (**Figure 4B**). Additionally, another important way of regulating autophagy by phase separation is that transcription factors affect gene expression through phase separation. TFEB (transcription factor EB), which is responsible for autophagy and lysosome biogenesis gene transcription (73), forms nuclear condensates and then colocalizes with MED1 puncta, thus participating in the activated expression of genes involved in the autophagic lysosomal pathway (74).

## Concluding remarks and future perspectives

Here, we summarize the discoveries of novel signaling mechanisms of phase separation in innate immune response during inflammation. Phase separation represents a new paradigm in hub signaling and provides several new perspectives on understanding biological systems such as precise immune regulation (75, 76). The formation of membraneless condensates driven by phase separation flexibly regulates innate immune signaling, in a spatiotemporal control pattern, including the innate immune pathways cGAS-STING, innate immune sensor NLRP6, autophagy-related factors p62/SQSTM1, NBR1, TFEB and RNA helicase DDX3X. In terms of dynamic spatiotemporal regulation, LLPS that mainly mediated by multivalent interactions through IDRs, is not only an important driving force for assembly of inflammasome and autophagic matrix, but also an induction pathway that activates innate sensors, host effectors and the transcriptional cascade, thereby enriching or separating intracellular components and enabling them to be rapidly and efficiently activation (65). Recently, increasing evidences have emerged that LLPS is involved in innate immune responses to viral infections. For example, SARS-CoV-2 nucleocapsid protein can form LLPS with viral genomic RNA to promote viral replication and assembly (77). The dimerization domain of SARS-CoV-2 nucleocapsid protein that is responsible for the LLPS, inhibits Lys63-linked polyubiquitination and MAVS aggregation, and thus blocks innate antiviral immune response (78). These studies provide a new framework for understanding the mechanisms by which viruses replicate and may aid in the development of antiviral drugs.

To date, the role of phase separation in immune signal transduction, such as cGAS-STING and inflammasome signaling, links with the regulation of pathogen recognition, protein complex assembly and organelle homeostasis leading to the activation of innate immune pathways have been demonstrated (75, 76, 79). The specific mechanisms underlying the dynamic characteristics of phase separation in innate immunity are still lacking. Research on the mechanisms of phase separation, especially the screening of key molecules that regulate phase separation, will provide a theoretical basis for the identification of effective targets. There might be important theoretical and practical significance for both physiological exploration and pathological function research of phase separation in regulation of innate immune response and inflammation-related diseases.

## Author contributions

All authors contributed to gathering of data, writing, editing, and revising of the manuscript. All authors contributed to the article and approved the submitted version.
